# Wear Estimation of DLC Films Based on Energy-Dissipation Analysis: A Molecular Dynamics Study

**DOI:** 10.3390/ma15030893

**Published:** 2022-01-25

**Authors:** Zhiyuan Yin, Hong Wu, Guangan Zhang, Chenzhong Mu, Lichun Bai

**Affiliations:** 1 Key Laboratory of Traffic Safety on Track, Ministry of Education, School of Traffic & Transportation Engineering, Central South University, Changsha 410075, China; yzy082@csu.edu.cn; 2 State Key Laboratory of Powder Metallurgy, Central South University, Changsha 410083, China; hwucsu@csu.edu.cn; 3 State Key Laboratory of Solid Lubrication, Lanzhou Institute of Chemical Physics, Chinese Academy of Sciences, Lanzhou 730000, China; gazhang@licp.cas.cn; 4 State Key Laboratory of Special Functional Waterproof Materials, Beijing Oriental Yuhong Waterproof Technology Co., Ltd., Beijing 100123, China; mucz@yuhong.com.cn; 5 State Key Laboratory for Strength and Vibration of Mechanical Structures, Xi’an Jiaotong University, Xi’an 710049, China

**Keywords:** energy-dissipation analysis, wear estimation, diamond-like carbon, molecular dynamics simulation

## Abstract

This study employs the energy-dissipation method to analyze the tribological behaviors of diamond-like carbon (DLC) films through molecular dynamics simulation. It is found that at small load and sliding velocity, the variation trend of average friction force is only dependent on the number of interface bonds (or contact area). However, at large load and sliding velocity, the friction mechanism is not only related to the number of interface bonds but also related to the presence of the transfer layer. The elastic–plastic deformation mainly occurs in the early sliding stage, and a part of the stored elastic potential energy is dissipated by plastic potential energy or internal frictional heat. After the sliding stabilization, over 95% of the total frictional energy is dissipated by thermal conduction, and the rest is mostly dissipated by wear. The increase in load, velocity, and temperature cause more frictional energy dissipated by elastic–plastic deformation, atomic motion, and elastic deformation instead of thermal conduction, respectively. Finally, the wear rate obtained in this work is the same order of magnitude as the experiment. Generally, this work provides an effective atomic-scale method to comprehensively analyze the microscopic wear mechanism of materials.

## 1. Introduction

In recent years, with the widespread use of precision machinery, people have paid more and more attention to improve the reliability and service lifetime of these machinery. Therefore, it is important to understand the tribological laws at the small scale, especially the nanoscale. 

Friction is a violent physical process that leads to the deformation and wear of materials. Energy-dissipation analysis, which has been mainly used in macroscopic experiments, is proved to be an effective method in studying wear [1,2,3,4,5,6,7,8]. Fouvry et al. found that the energy consumed in the friction process is proportional to the wear volume in the study of fretting wear [1,2]. A similar result was observed by Ramalho et al. [3]. In their later research on fretting wear, the relationship between the total energy dissipated and the wear mechanism (plastic deformation, structural transformation, stable wear state, etc.) was established [4,5]. Since the macroscale wear is mainly caused by plastic deformation and fatigue, under certain working conditions, the energy dissipated per unit of wear volume can be calculated from the friction coefficient and the mechanical properties of the material (hardness, fatigue strength, etc.) [6]. Based on this point of view, many researchers have established a direct correlation between the total wear volume and the test conditions as well as the mechanical properties of materials [7,8,9,10]. 

Energy dissipation is an important phenomenon in the friction and wear process. In recent years, the application of energy dissipation analysis in nano-scale tribology has mainly focused on the analysis of friction mechanism [11,12,13,14,15,16]. According to the Tomlinson model, the energy dissipation in atomic friction is induced by mechanical instability, which can cause viscous slip motion and atomic motion [11]. The Frenkel–Kontorova model shows that the structural comparability between two contact surfaces highly affects the magnitude of friction and energy dissipation, and it can achieve super-lubrication in completely disproportionate systems [12,14]. The Cobblestone model found that sliding consists of a series of surface separations and proximities, and the energy loss in the surface separation method determines the friction [15,16]. However, the above models fail to establish the direction correlation between the energy dissipation and wear volume. Since the proportion of energy consumed by various energy-dissipation methods (such as elastic–plastic deformation, thermal diffusion, phase change, wear etc.) is difficult to measure in experiments, this hinders the application of energy-dissipation analysis in nano-wear.

Diamond-like carbon (DLC) films have excellent mechanical and friction properties and have been widely used to improve the wear resistance of components at micro, meso, and macro scales [17,18,19,20]. Many molecular dynamics (MD) simulations have shown that the tribological and mechanical behaviors of DLC films are very sensitive to the test conditions (such as load, velocity, temperature, surface modification) [17,18]. Moreover, DLC films have a graphitization phase (sp^3^-sp^2^) transition during the friction process [19,20]. These phenomena enable that the wear mechanisms of DLC films can reflect the influence of key factors in the nano-wear process. Therefore, DLC films are ideal materials for studying the principal laws of nano-wear and have been employed in many previous studies [21,22,23]. 

In order to establish the correlation between the wear and energy dissipation, this work investigates the nano-wear mechanism of DLC films by employing the energy-dissipation analysis method via MD simulation. The considerations are given to the effects of testing conditions (load, velocity, and temperature). 

## 2. Modeling

**Figure 1:**
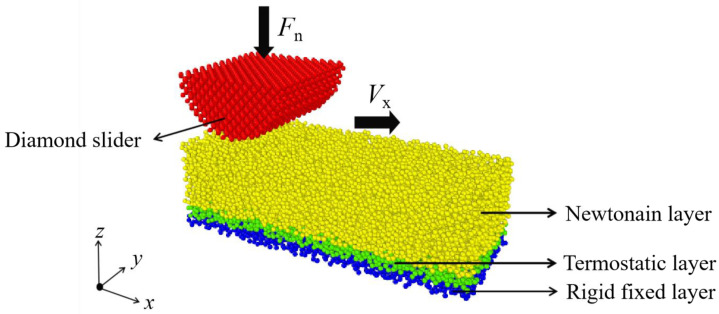
Schematic illustration of MD friction model.

Figure 1 shows that the simulation model is composed of a diamond slider and a diamond-like carbon film. A load *F*_n_ along the *z*-direction and a velocity *V*_x_ along the *x*-direction are applied to the diamond slider to cause the wear of the DLC film. The diamond slider that has a semi-cylindrical shape is located above the DLC film along the *z*-direction. In the *x*-*z* plane, this slider is a semicircle with a radius of 20 Å. Moreover, along the *y*-direction, the dimension of the slider is consistent with that of the DLC film. In order to simplify the wear analysis, the diamond slider is kept as a rigid body to keep its structure undeformed. The dimensions of DLC film are 100 × 40 × 30 Å^3^. Along the *z*-direction, the DLC film below is divided into three layers with different functions. The bottom layer with a thickness of 3 Å is kept as a rigid body to prevent the relative sliding of the film. The layer above on the rigid one with a thickness of 3 Å is coupled to a Nose–Hoover thermostat to dissipate the thermal energy and maintain a stable temperature. The rest that contains the Newtonian atoms is the top layer, which is used to contact with the diamond slider. According to Newton’s second law, atoms in the Newtonian layer are unconstrained and can move freely under the forces of the surrounding atoms. The periodic boundary conditions of the system are set along both the *x* and *y*-directions. 

The melting–quenching procedure is a reasonable way to obtain DLC film. The initial model with diamond structures is generated at 300 K in a standard NVT ensemble. The boundaries of such model are periodic in all directions. Afterwards, the system temperature rapidly rises above the melting point of diamond (10,000 K), and the sample is equilibrated for about 20 ps. Then, in order to properly relax the model structure, the system temperature is cooled to 300 K at a rate of 1000 K/ps. Finally, the structure is relaxed at 300 K in the isothermal–isobaric ensembles (NPT) to eliminate its residual stress. Finally, the sp^2^-to-sp^3^ ratio of the final model is 0.56 with the system density of 3.01 g/cm^3^ (the radial distribution function image of DLC film is shown in Figure S1 of the Supporting Materials). More details regarding such a procedure may refer to previous studies [18,24]. 

Before the sliding starts, a load *F*_n_ (78, 196, 392, or 588 nN) is applied on the diamond slider. The loading procedure lasts for about 50 ps to stabilize the DLC film under external loading. After the stabilization of the contact, the relative sliding between the slider and the DLC film is realized by applying the velocity *V*_x_ (0.2, 0.4, 0.5, or 1 Å/ps) to such a slider. The influence of temperature *T* on this simulation is considered by setting different temperatures (200, 300, or 400 K) of the thermostat during the loading procedure. The base conditions are set as *F*_n_ = 392 nN, *V*_x_ = 1 Å/ps, and *T* = 300 K. When investigating the effect of one of these three factors, the others are kept unchanged. All simulations are conducted via the large-scale atomic/molecular massively parallel simulator (LAMMPS) [25]. The interactions of the C-C atoms are obtained by using Tersoff potential, which can describe the dynamic physical process of various types of carbon materials [26]. The timestep of simulation is set to 1 fs, and the sliding distance (*L*) in each simulation is kept at 1000 Å. The visualization of the simulation results is conducted via the OVITO software (Version 3.3.5) [27].

The friction force (*F*_f_) is obtained through summing up the tangential force of atoms in the diamond slider along the *x*-direction. *F*_a_ is defined as the average friction force of the entire sliding process. The number of nearest neighbors of atoms within a cutoff length of 2.0 Å for C-C pairs are defined as their coordination numbers, which are used to characterize the hybridizations states of carbon atoms [28]. The threefold and fourfold carbon atoms are regarded as sp^2^ and sp^3^ bonded, respectively [18]. 

The definition of worn atoms follows the standard in previous research by MD simulations. Zhong et al. defined atoms removed from the substrates as worn atoms [29]. However, this definition is more suitable for the macroscopic experiment to predict the wear volume. The definition above ignored such atoms. Therefore, it is more appropriate to qualify worn atoms by evaluating their displacement. In amorphous solids, atoms that escape from the bond or surround of their nearest neighbor with displacement of at least two bond lengths are recognized as worn atoms, which has been used as a qualification for worn atoms in this study [30]. Since the DLC film is amorphous solid and the maximum bond length in this film is about 2 Å, its atoms with displacements exceeding 4 Å are identified as worn atoms [31]. It is worth mentioning that atomic coordinates are the basis for judging the displacement distance of atoms in this work. However, atoms close to the boundary are affected by the periodicity of the system, and artifacts can be introduced if periodic boundary conditions are not properly taken into account. Therefore, the atoms at the edge of the model are not considered. Meanwhile, the calculation results of various types of energy are correspondingly taken into account. It needs to be stated that the above settings achieve ideal conditions; that is, they are only suitable for estimation rather than accurate calculation. The wear energy (*E*_w_) is obtained by calculating the difference between the total potential energy of the worn atoms and their initial potential energy.

Based on the above identification of worn atoms during the wear process, the remaining atoms in the DLC film are defined as non-worn atoms. Among these non-worn atoms, the atoms whose coordination number has changed relative to their initial value are identified as plastically deformed atoms. In the same way, atoms whose coordination number has not changed are identified as elastically deformed atoms. The plastic potentials energy *E*_p_ (and elastic potentials energy *E*_e_) are obtained by calculating the difference between the total potential energy of the plastically deformed atoms (as well as elastically deformed atoms) and their initial potential energy. The kinetic energy (*E*_k_) is obtained by calculating the difference between the total kinetic energy of non-worn atoms and their initial kinetic energy. The frictional energy (*E*_f_) can be obtained with the form [32]

(1) $$E_{f}\;=\;V\int_{0}^{t_{\exp}}F(t)\mathrm{dt}$$

where *t*_exp_ is the sliding time, *F*(*t*) is the friction force as a function of time, and *V* is the sliding velocity. *E*_f_ is the total energy consumed by sliding. 

The energy-dissipation analysis method divides the dissipation forms of the friction process into heat dissipation, material deformation dissipation, and wear dissipation [33,34]. The deformation dissipation includes elastic potential energy, plastic potential energy, and kinetic energy. Therefore, Equation (1) can be also expressed as

(2) $$E_{f}\;=\;E_{w}+E_{p}+E_{e}+E_{k}+E_{t}$$

where the energy dissipated by thermal conduction (*E*_t_) can be obtained by substituting the value of the energy form calculated above into Equation (2). 

## 3. Results

### 3.1. Friction Interface and Friction Behavior

**Figure 2:**
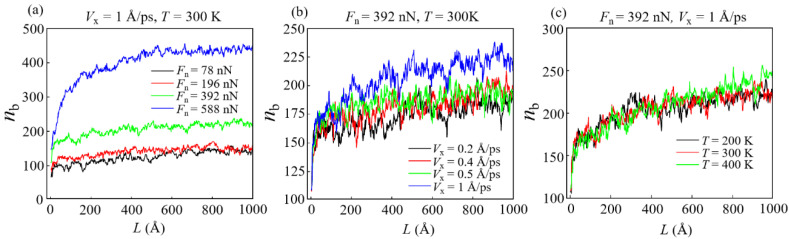
Number of bonds *n*_b_ between the DLC film and the slider with different conditions: (**a**) *V*_x_ = 1 Å/ps, *T* = 300 K; (**b**) *F*_n_ = 392 nN, *T* = 300 K; (**c**) *F*_n_ = 392 nN, *V*_x_ = 1 Å/ps.

As the slide progresses, a large amount of bonds are formed between the DLC film and the diamond slider. Generally, the formation of these bonds is accompanied by the generation of worn atoms. Furthermore, the number of such bonds can be used to determine the size of the contact area, contact structure, the degree of wear, and the wear mechanism. Figure 2 shows the influence of the number of bonds (*n*_b_) between the DLC film and the slider in the entire sliding process with different conditions. *n*_b_ increases rapidly in a short period of time at the beginning of sliding. Then, it reaches a stable stage and increases slowly, which indicates the beginning of the stable sliding stage. It is found that *n*_b_ increases with *F*_n_, *V*_x_, and *T*. Among them, *F*_n_ has the most significant effect on *n*_b_, which is consistent with previous research results [23,35,36]. It is worth noticing that under different *T* conditions, *n*_b_ only began to show differences in the middle and later stages of sliding (Figure 2c). 

**Figure 3:**
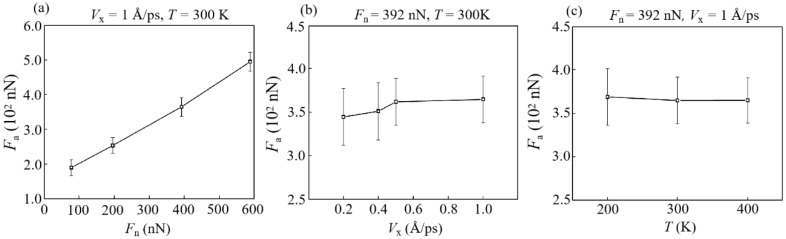
Effect of different conditions on the average friction force *F*_a_: (**a**) *V*_x_ = 1 Å/ps, *T* = 300 K; (**b**) *F*_n_ = 392 nN, *T* = 300 K; (**c**) *F*_n_ = 392 nN, *V*_x_ = 1 Å/ps.

In previous MD studies of nano-wear, the friction force commonly depends on the number of bonds between the contact interfaces [36,37,38]. The variation trend of average friction force *F*_a_ under different conditions is shown in Figure 3, and the change of friction force *F*_f_ with *L* under different conditions is shown in Figure S2 of the Supporting Materials. Obviously, a high *n*_b_ means a large contact area and a high degree of wear, and it can cause more atoms (that is, worn atoms) moving with the diamond slider during the sliding [23]. Therefore, a high *n*_b_ corresponds to a high *F*_a_. Figure 3a shows that *F*_a_ increases as a linear function of *F*_n_. However, *n*_b_ increases almost as a quadratic function of *F*_n_ (Figure 2a shows that *F*_n_ values of 78, 196, 392, and 588 nN correspond to average *n*_b_ values of about 124.8, 141, 205.3, and 399, respectively). This means that due to the complexity of the wear mechanism, under certain working conditions (such as high loads), the friction force is not completely determined by the number of interface bonds (or contact area). Similar considerations also occur in sliding velocity conditions (Figure 2b or Figure 3b); that is, the function of *n*_b_ with *F*_n_ is different from that of *F*_a_ with *F*_n_. Furthermore, as *T* increases, *F*_a_ decreases slightly instead. These phenomena can be explained by the transformation of the microstructures at the contact interface [39]. 

**Figure 4:**
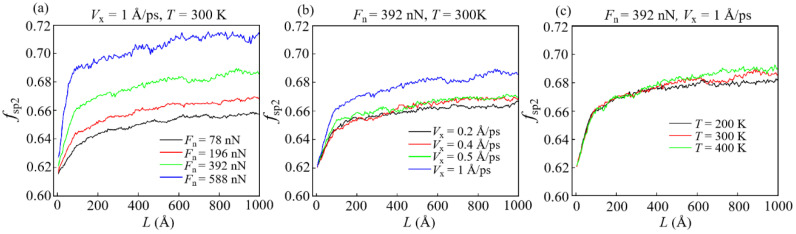
Influence of different conditions on the fraction of sp^2^-C atoms *f*_sp2_ of DLC films: (**a**) *V*_x_ = 1 Å/ps, *T* = 300 K; (**b**) *F*_n_ = 392 nN, *T* = 300 K; (**c**) *F*_n_ = 392 nN, *V*_x_ = 1 Å/ps.

The fraction of sp^2^-C atoms (*f*_sp2_) of the DLC film is usually used to characterize its microstructures, as given in Figure 4. It is noticed that a large *F*_n_ and a large *V*_x_ can increase the *f*_sp2_ and cause graphitization of the DLC film. In this case, a high *f*_sp2_ can reduce the strength limit of DLC film and induce its easy-shear properties at the contact interface [40,41]. Such a reduction is commonly due to the formation of a transfer layer at the contact interface. This demonstrates that besides the one dominated by the contact area, there is another friction mechanism in this sliding process. Since the atoms that form the transfer layer are attached under the slider and they slide with it [35], such a layer is defined as all atoms whose velocity is almost equal to that of the slider at any sliding time. It is found that the resulting transfer layer is commonly sp^2^-dominant, which conforms to previous research on the transfer layer (the sp^2^ ratio of the transfer layer is shown in Figure S3 of the Supporting Materials) [35,42]. 

**Figure 5:**
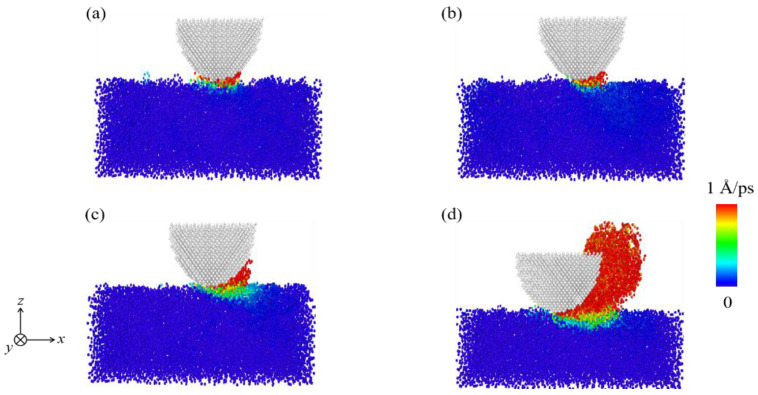
Schematic diagram of the transport layer at the stable sliding stage with (**a**) *F*_n_ = 78 nN, (**b**) *F*_n_ = 196 nN, (**c**) *F*_n_ = 392 nN, and (**d**) *F*_n_ = 588 nN.

Figure 5 is a schematic diagram of the transfer layer under load conditions (the videos of the sliding track of the transfer layer are shown in Videos S1–S4 of the Supporting Materials). From the definition above, it follows that the formation of such a layer prevents the direct contact between the film and the slider to a certain extent. The lubricant-like effect of this layer helps to reduce *F*_a_. Meanwhile, the increase in *f*_sp2_ commonly means the formation of a thicker transfer layer, which has a more significant impact on the reduction of *F*_a_. As a result, at small *F*_n_ and *V*_x_, the variation trend of *F*_a_ only depends on the number of interface bonds (or contact area). However, at a large *F*_n_ and *V*_x_, the friction mechanism is not only related to the number of interface bonds but also related to the presence of the transfer layer. The combined action of the two mechanisms leads to *n*_b_ and *F*_a_ increase as different functions of *F*_n_. 

In addition, the increase in *T* can soften the microstructure of DLC film and degrade its mechanical properties [43]. Under different *T* conditions, the change of *f*_sp2_ is similar to that of *n*_b_ (Figure 2c or Figure 4c). It is found that *T* values of 200, 300, and 400 K correspond to the average number of atoms in the transfer layer during the entire sliding process of 107.2, 116.4, and 119.5, respectively. It means that in the middle and late stages of sliding, the temperature of the contact interface rises to soften the DLC film, which makes it easier to form a transfer layer between the DLC film and the slider. Therefore, when *T* increases, the reason for the slight decrease in *F*_a_ is that the easy-shear performance of DLC film induces the formation of a transfer layer. 

### 3.2. Analysis of Energy Forms

**Figure 6:**
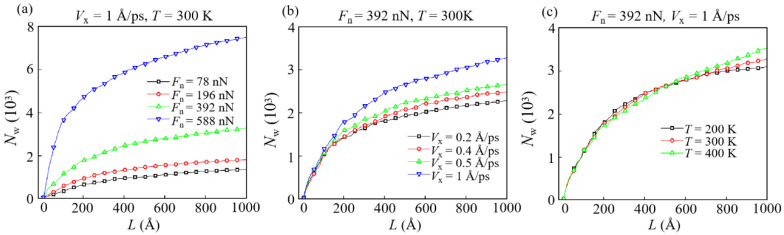
The evolution of the number of worn atoms *N*_w_ with different conditions: (**a**) *V*_x_ = 1 Å/ps, *T* = 300 K; (**b**) *F*_n_ = 392 nN, *T* = 300 K; (**c**) *F*_n_ = 392 nN, *V*_x_ = 1 Å/ps.

**Figure 7:**
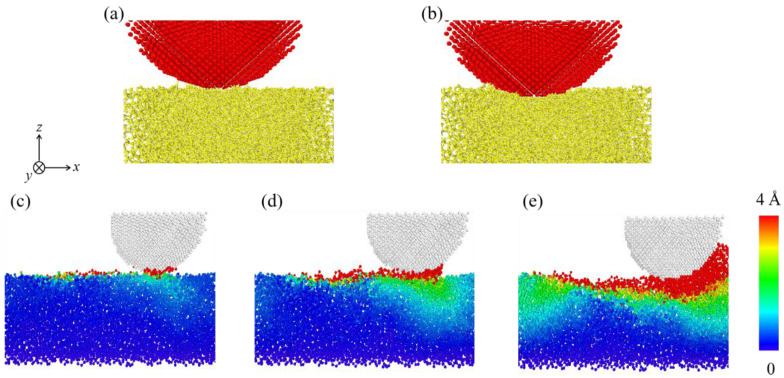
The contact configuration between the DLC film and the diamond slider with (**a**) *F*_n_ = 78 nN and (**b**) *F*_n_ = 588 nN when *L* = 0; contour of atom displacement with (**c**) *F*_n_ = 78 nN, (**d**) *F*_n_ = 392 nN, and (**e**) *F*_n_ = 588 nN when *L* = 50 Å. The color of the atom indicates its displacement distance.

The number of worn atoms (*N*_w_) in the DLC film that represents the worn volume is also influenced by different conditions, as shown in Figure 6. The change of *N*_w_ with conditions is highly similar to that of *n*_b_, which represents the actual contact area. It is worth noting that for *F*_n_ = 588 nN, a large number of atoms belonging to the DLC film are worn off (Figure 5d or Figure 6a), which is determined by the contact configuration and sliding mode between the DLC film and the diamond slider. The evolution of their contact interfaces under different *F*_n_ and at different moments is shown in Figure 7. Under a large *F*_n_ (588 nN), the bottom of the slider penetrates the DLC film (Figure 7b), resulting in the appearance of a chip in front of the slider during the sliding (Figure 5d or Figure 7e). Such an appearance is accompanied by a large increase in *N*_w_. This is due to the fact that a large *F*_n_ highly changes the contact configuration, affecting the wear form between the slider and the film (Figure 7a,b). 

The transition of the wear form caused by the variation of the load has been confirmed in many experiments [44,45,46]. Yoon et al. reported that the friction force of DLC film was mainly affected by adhesion at small loads, and elastic deformations happen in such a film [47]. Smerdova et al. indicated that as the worn area became larger, other wear mechanisms interfered [44]. Yang et al. used MD simulations to demonstrate that wear can exhibit two forms of local atom shedding wear and large-scale plastic wear [48]. Such two forms can also be observed in these simulations. At a lower *F*_n_, part of the atoms is shed off the DLC film and slide with the slider (Figure 7d). However, in the case of *F*_n_ = 588 nN, the wear form is transformed from atoms shed locally to large-scale plastic wear, which leads to a large increase in *N*_w_ (Figure 7e and Video S4 of the Supporting Materials). For this reason, the average contact stress is obtained by dividing the applied load *F*_n_ by the average contact area between the slider and the DLC film after the sliding is stabilized. The results show that *F*_n_ values of 78, 196, 392, and 588 nN correspond to average contact stresses of about 13.2, 30.6, 46.7, and 63.9 GPa, respectively. The hardness limit of this film was found to be about 60.8 GPa in the nanoindentation test at room temperature (the nanoindentation curve is shown in Figure S4 of the Supporting Materials). Moreover, the increase in temperature of the contact interface can significantly reduce the strength of the DLC film during the sliding process, resulting in a further reduction in the hardness limit of this film. Therefore, for *F*_n_ = 588 nN, such a reduction leads to the structural degradation of the DLC films and a large increase in *N*_w_. 

When *T* increases, the total *N*_w_ becomes larger, but there is a slight decrease in the early and middle stage of sliding (about *L* = 200–500 Å), which will be discussed later. This reason is more appropriately explained by analyzing the form of energy dissipation during the sliding process. Moreover, as the adhesion and worn area increase, more friction energy is dissipated in the form of surface heating or heat conversion, fatigue, and adhesive wear in the friction process [44]. Therefore, it is important to determine the proportion of energy consumed by different energy dissipation in the nano-friction process to understand its wear mechanism. 

**Figure 8:**
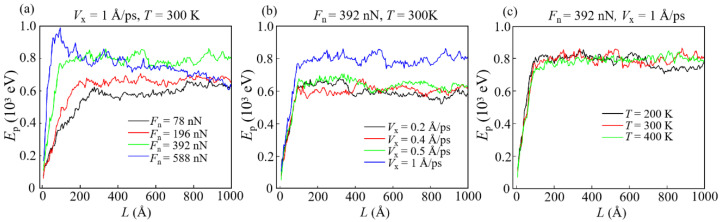
The evolution of plastic potential *E*_p_ under different conditions: (**a**) *V*_x_ = 1 Å/ps, *T* = 300 K; (**b**) *F*_n_ = 392 nN, *T* = 300 K; (**c**) *F*_n_ = 392 nN, *V*_x_ = 1 Å/ps.

**Figure 9:**
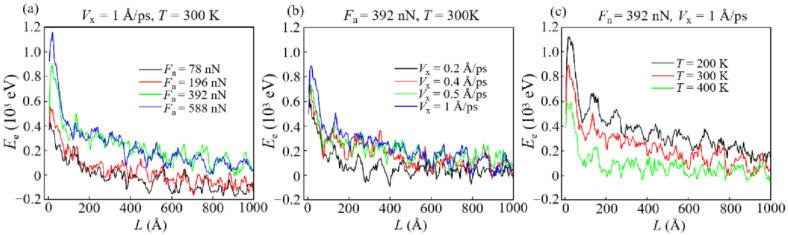
The evolution of elastic potential energy *E*_e_ under different conditions: (**a**) *V*_x_ = 1 Å/ps, *T* = 300 K; (**b**) *F*_n_ = 392 nN, *T* = 300 K; (**c**) *F*_n_ = 392 nN, *V*_x_ = 1 Å/ps.

**Figure 10:**
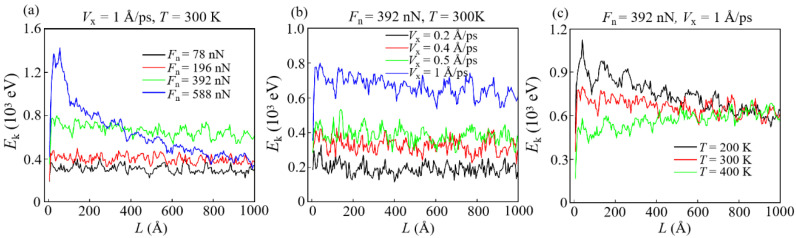
The evolution of the kinetic energy *E*_k_ under different conditions: (**a**) *V*_x_ = 1 Å/ps, *T* = 300 K; (**b**) *F*_n_ = 392 nN, *T* = 300 K; (**c**) *F*_n_ = 392 nN, *V*_x_ = 1 Å/ps.

There is a common belief that the wear particles fall off during the friction process, and the potential energy accumulated inside such particles is defined as the energy dissipated by wear [49]. Since the evolutions of wear energy *E*_w_ are highly consistent with that of *N*_w_, the evolution of *E*_w_ calculated by the above definition under different conditions is only shown in Figure S5 of the Supporting Materials. Then, the energy evolutions realized by deformation and atomic motion during the wear process are classified by the dissipation mode, and the result is shown in Figure 8, Figure 9 and Figure 10. 

It is found that as the sliding starts, the plastic potential energy (*E*_p_) first increases rapidly to a maximum and then stabilizes around a certain value (Figure 8). This certifies that plastic deformations occur in the early stage of sliding, and a high *n*_b_ generally corresponds to faster and more severe deformations. Since the deformation of the contact interface is the most severe in the early stage of sliding (Figure 7c–e), the increase in plastic potential energy is caused by the plastic deformation that occurs at the contact interface. Specially, at a large *F*_n_ (588 nN), *E*_p_ decreases after it reaches a maximum value instead of remaining stable (Figure 8a). This decrease is due to the degradation of the structure of DLC film caused by the excessive load (contact stress exceeding the hardness limit of DLC film), leading to a large number of atoms to be identified as worn atoms instead of plastically deformed ones. 

Unlike *E*_p_, the elastic potential energy (*E*_e_) reaches the maximum in a very short time and then gradually decreases with the increase in *L* (Figure 9). The increase in *E*_e_ at the beginning of sliding means that the contact stress in the elastically deformed area is not enough to generate the plastic deformation, and thus, the elastic potential energy is stored as strain energy in carbon networks [50]. Then, the decrease in *E*_e_ reveals that a part of the stored strain energy is converted to *E*_p_ or internal frictional heat. It is found that a reduction of *T* is related to an increase in *E*_e_ in the DLC film (Figure 9c). This correspondence is attributed to the fact that DLC films have higher mechanical strength at a lower *T*, making it more difficult for the sp^3^-C atoms of the film to be converted into sp^2^-C atoms (Figure 4c), which thus results in an increase in *E*_e_. 

The kinetic energy (*E*_k_) of non-worn atoms usually increases rapidly and stabilizes after the start of sliding (Figure 10). For *F*_n_ = 588 nN, the reason for the decrease in *E*_k_ is the same as that for the decrease in *E*_p_. *E*_k_ represents the active level of atomic movement in the DLC film. A larger *E*_k_ means that these atoms have a higher probability of transforming into worn atoms. Simultaneously, it can be found that in the early and middle period of sliding (*L* < about 500 Å), *E*_k_ at a lower *T* is higher than that at a higher *T* (Figure 10c). Therefore, during this period, *N*_w_ at a lower *T* is higher than that at a higher *T* (Figure 6c). Moreover, in the middle and late stages of sliding (*L* > about 500 Å), the high temperature induces the low mechanical strength and high *f*_sp2_ of DLC film, causing a large number atoms to form the transfer layer (Figure 4c). This formation leads to *N*_w_ at higher temperatures larger than that at lower temperatures (Figure 6c). 

Clearly, in the case of the same wear form, a high *F*_a_ always corresponds to a large summation of *E*_e_ and *E*_p_. This fact clarifies the statement mentioned above in a quantitative way that high contact stress and high contact interface adhesion can cause the large-scale plastic and elastic deformations. 

### 3.3. Analysis of Energy Dissipation

**Figure 11:**
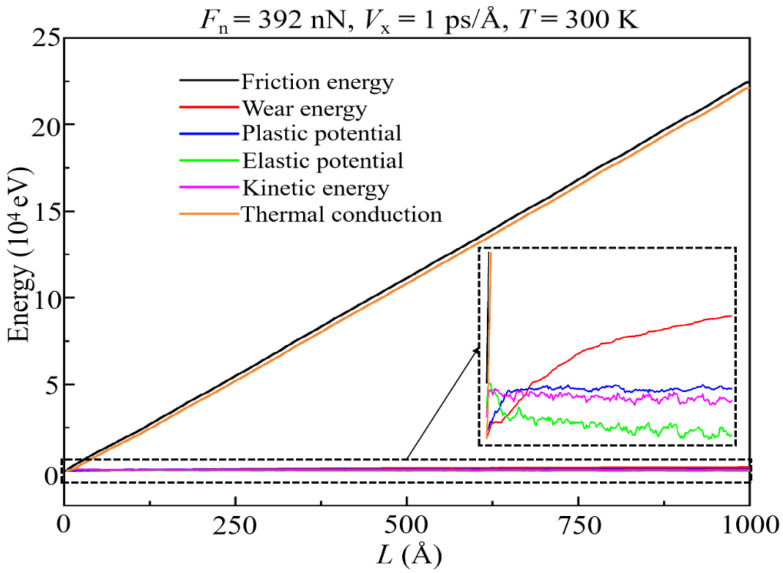
The evolution of various energy dissipation forms with sliding distance.

Energy dissipation is an important phenomenon in the friction process. It is an effective method to understand the nano-wear mechanism by dividing the energy dissipation forms and analyzing their changes in the friction process [2,4,5]. Based on the above analysis and previous studies, the total frictional energy (*E*_f_) consumed in the sliding process is dissipated by thermal conduction (*E*_t_), elastic (*E*_e_) and plastic potential (*E*_p_), wear energy (*E*_w_), and kinetic energy (*E*_k_) [33,34,51,52]. A typical example is given by studying various types of dissipated energy changes with sliding distance under reference operating conditions (*F*_n_ = 392 nN, *V*_x_ = 1 Å/ps, *T* = 300 K), as shown in Figure 11. The same type of diagrams under the remaining working conditions are shown in Figures S7–S9. It can be seen that most of *E*_f_ are dissipated in the form of *E*_t_. This is due to the friction force and the relative sliding causing the generation of a large amount of heat at the sliding interface that is majorly dissipated by the thermostat layer. Furthermore, the stability of *E*_e_ and *E*_p_ with sliding distance indicates that the deformation of the DLC film is reduced, and the sliding enters a stable stage. After this stage, *E*_f_ is mainly dissipated in the form of *E*_t_ and a small amount of *E*_w_. 

The total amount of energy dissipation (i.e., *E*_f_) under different conditions is shown in Figure S6 of Supporting Materials. It can be seen that *E*_f_ has a linear relationship with the sliding distance. In addition, Jahangiri et al. pointed out that a high friction force caused by a high *F*_n_ is the dominant reason for the increase in *E*_f_, which can also be verified in this simulation [53]. 

**Figure 12:**
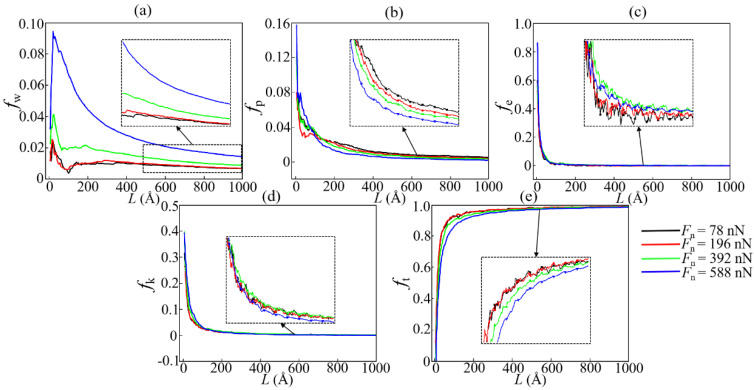
Evolution of the proportion of various energy dissipation forms under different *F*_n_ at *V*_x_ = 1 Å/ps, *T* = 300 K: (**a**) *f*_w_; (**b**) *f*_p_; (**c**) *f*_e_; (**d**) *f*_k_; (**e**) *f*_t_.

For the purpose of more intuitively presenting the evolution of various energy dissipation forms in the wear process, the ratios of the wear energy (*f*_w_), plastic potential energy (*f*_p_), elastic potential energy (*f*_e_), kinetic energy (*f*_k_), and energy dissipated by thermal conduction (*f*_t_) to *E*_f_ under different conditions are shown in Figure 12 (and Figures S10 and S11 of the Supporting Materials). It can be seen that the proportion of energy consumed by plastic deformation and wear during most of the sliding time does not exceed 10%, which confirms the experimental predictions [33,54]. It is noticed that the presence of the transfer layer is the critical point for the transition of the sliding mode (about *L* = 100–200 Å). Before the formation of this layer, especially at the beginning of sliding, the energy dissipated by wear, deformation, and atomic motion both occupy part of *E*_f_. This is due to the DLC film being squeezed by the slider and undergoing elastic–plastic deformation and a lot of wear in this stage. Meanwhile, the thermal conduction induced by high temperature at the sliding interface leads to the rapid increase in *f*_t_, thereby resulting in the rapid decrease in *f*_w_, *f*_p_, *f*_e_, and *f*_k_. After the formation of the transfer layer, friction sliding mainly happens inside such a layer, resulting in that the DLC films are hardly deformed. Therefore, in the stable wear state, *E*_f_ is mostly dissipated by heat diffusion (*f*_t_ more than 95%), and the rest is mostly dissipated by wear. 

With reference to Figure 3, Figure 12, Figures S10 and S11, it can be found that working conditions with higher friction force always correspond to that with lower *f*_t_. This is due to the high friction force induced by high load leading to more *E*_f_ being dissipated by wear and elastic–plastic deformation (Figure 12). However, the reasons for the decrease in *f*_t_ due to the varieties of *V*_x_ and *T* are not the same as those for *F*_n_. When *V*_x_ increase, besides the above reason, high *V*_x_ also causes more *E*_f_ to be dissipated by atomic motion (Figure S10). When *T* decreases, the reduction of *f*_t_ is due to a lower *T* causing the high elastic modulus and mechanical strength of the DLC film, and thus, more energy is stored as strain energy, which is mentioned above. As a result, at a lower *T*, more *E*_f_ is dissipated by elastic deformation and atomic motion, thereby reducing *f*_t_ (Figure S11). 

It has been reported that some models relate the wear rate (*k*_w_) with energy dissipation [44,55]. This can be used to calculate *k*_w_, which can quantify the relationship between the wear volume (*V*_w_) and the total energy. The direct relationship between *V*_w_ and test conditions or friction force (*F*_f_) can be established by such a method. Since *F*_n_ has the most significant impact on wear in the experiment, we take this condition as an example to estimate *k*_w_ of the material simulated in this study. Generally, *k*_w_ is calculated considering the steady phase only. Therefore, all values selected below (*N*_w_, *F*_f_, *F*_w_, and *E*_f_) are stable values at the end of the simulation. The equation that defines *V*_w_ in experiments is

(3) $$V_{w}\;=\;k_{w}\cdot F_{N}\cdot L$$

where *k*_w_ is the material wear rate, *F*_N_ is the applied load, and *L* is the sliding distance. In this study, *V*_w_ can be given by

(4) $$V_{w}\;=\;\frac{N_{w}\cdot m_{c}}{\rho }$$

where *N*_w_ is the number of worn atoms, and values of *F*_n_ of 78, 196, 392, and 588 nN are 1379, 1812, 3276, and 7493, respectively. *m*_c_ is the mass of carbon atoms, and its value is 1.99 × 10^−23^ g, *ρ* is the density of the DLC film, and its value in this work is 3.01 g/cm^3^. In the experiment, the wear energy (*E*_w_) is given by [8]

(5) $$E_{w}\;=\;f_{w}\cdot F_{N}\cdot L\cdot \mu$$

where *f*_w_ is the proportion of the wear energy consumed to the total frictional energy, and their values are 0.67%, 0.68%, 0.88%, and 1.44% corresponding to *F*_n_ conditions of 78, 196, 392, and 588 nN, respectively. Similarly, the values of *E*_w_ under different *F*_n_ conditions are 870.4, 1063.5, 1970.4, and 4386.7 eV, respectively. *μ* is the friction coefficient, which can be obtained by calculating the ratio of *F*_a_ to *F*_n_. Similarly, the values of *μ* under different *F*_n_ conditions are 2.24, 1.29, 0.93, and 0.84, respectively. Substituting Equations (4) and (5) into Equation (3), it can be written as

(6) $$k_{w}\;=\;\frac{\mu \cdot f_{w}\cdot N_{w}\cdot m_{c}}{E_{w}\cdot \rho }$$

*k*_w_ under different loads calculated according to the above formula is shown in Table 1. It is noticed that *k*_w_ decreases with the increase in *F*_n_. However, a larger *k*_w_ under the load condition of *F*_n_ = 588 nN is due to a high *N*_w_ caused by the degraded DLC film structure, which has been mentioned above. It has been reported that for a non-hydrogenated DLC, higher coefficient of friction (COF) values and wear rates were observed under vacuum compared to ambient air [56,57]. Since the simulations in this work are also performed in a vacuum environment and with a non-hydrogenated DLC film, the comparison with experimental results should be sensible. The higher wear rate calculated in this study indicates that MD simulation can estimate *k*_w_, and it also proves that the energy dissipation analysis method is effective. 

## 4. Discussions

The effect of the sliding velocity on the friction behavior of DLC films has been discussed in many experiments and MD simulations [18,24,35,40,58,59,60]. In these papers, friction force reduces with the increasing velocity, which is inconsistent with the results observed in this article. Such a reduction is commonly due to the fact that the high temperature induced by the high sliding velocity softens the DLC film, and thus, its elastic limit is reduced [24]. Simultaneously, the high temperature causes the graphitized phase transition of the DLC film to form an easy-to-shear transfer layer, also leading to the reduction of friction force [18]. However, the selection of different initial conditions in MD simulations has a significant influence on the results. For example, Bai et al. studied the wear of a diamond tip and DLC film through MD simulations [35]. The selection of a sliding velocity (>1 Å/ps), potential function, and the shape of the initial model in their simulations are different from this study. 

Moreover, within the sliding velocity range selected in this work, the increase in *V*_x_ leads to more energy being dissipated through wear and plastic deformation (Figure 12a,b), indicating that the friction mechanism is dominated by adhesion (or contact area) rather than structural changes (such as the formation of a thicker transfer layer). Therefore, the friction force increases with the increase in *V*_x_. It is worth mentioning that due to the significant influence of *F*_n_ on the wear mode, if *F*_n_ is set to values other than those in the cases of various *V*_x_, the correlation between the friction force and *V*_x_ may be completely different. This inference needs to be verified in future MD simulations. 

The present work proposes an effectively approach to analyze the wear behaviors of DLC film. Miyake et al. studied the influence of different environmental temperatures and found that a high temperature can significantly increase the nano-wear volume of the DLC film [61]. The present study explains in a quantitative way that the critical reason for such experimental observation is the degradation of the mechanical strength of the DLC film. Furthermore, Jahangiri et al. found linear relationships between the energy dissipation and wear distance as well as between wear volume and dissipated energy through a composite material wear experiment [53]. This linear relationship is also confirmed in this simulation. However, although some experimental equipment (such as AFM) can measure the wear volume, the data of other energy dissipation forms is still difficult to measure accurately due to the complexity of nano-wear and the limitation of such an equipment. Therefore, it is evident that the MD approach is an ideal method to overcome such difficulty.

In this work, the effects of atomic motion, thermo-elasticity of material, and viscoelastic relaxation on wear are considered. Meanwhile, this work clearly presents the dynamic change process of various energy proportions in the sliding process. This not only helps to establish the intrinsic relationship between wear and test conditions, material properties, and friction force, but also provides an effective method for solving the problem in experiments in which various types of dissipated energy cannot be quantitatively calculated.

## 5. Conclusions

The influence of different conditions on the nanotribological wear of DLC film is investigated through MD simulations. The average friction force *F*_a_ increases with the load *F*_n_ and sliding velocity *V*_x_. This increase is due to the high adhesion induced by the large number of bonds *n*_b_. However, *F*_a_ increases as a linear function of *F*_n_ but *n*_b_ increases almost as a quadratic function of *F*_n_, which is due to the increase in the fraction of sp^2^-C atoms *f*_sp2_ and the presence of the transfer layer at the contact interface. Specially, *N*_w_ increase highly at a large *F*_n_, which is due to the fact that high contact interface adhesion in this case can cause the wear form to transit from local atom shedding to large-scale plastic wear. *F*_a_ increases slightly with decreasing temperature *T*, which is because the DLC films have lower mechanical strength at a higher *T*. Meanwhile, such a lower mechanical strength of the DLC film causes more atoms to form the transfer layer and thereby leads to the increase in *N*_w_.

The effects of different energy dissipation forms on the wear mechanism of DLC films are studied in detail. The total frictional energy (*E*_f_) consumed is dissipated by thermal conduction (*E*_t_), elastic (*E*_e_) and plastic potential energy (*E*_p_), wear energy (*E*_w_), and kinetic energy (*E*_k_) in the present study. The proportions of the above energy forms are represented by *f*_t_, *f*_w_, *f*_p_, *f*_e_, and *f*_k_, respectively. It is found that *E*_f_ has a linear relationship with *L*. The elastic–plastic deformation mainly occurs in the early stage of sliding, and a part of the stored elastic potential energy is dissipated by converting to plastic potential energy or internal frictional heat. After the stabilization of sliding, since shear force and relative sliding cause a large amount of heat to be generated, most of *E*_f_ is dissipated in the form of *E*_t_ (more than 95%), and the rest is mostly dissipated by wear. Moreover, such generation is the reason for the rapid increase in *f*_t_ and the rapid decrease in *f*_w_, *f*_p_, *f*_e_, and *f*_k_. Working conditions with higher *F*_a_ always correspond to the reduction of *f*_t_. This is due to more *E*_f_ being dissipated by wear and elastic–plastic deformation (under varied load and velocity conditions), atomic motion (under diverse velocity and temperature conditions), and elastic deformation (under different temperature conditions). Finally, the wear rate *k*_w_ obtained in this work is the same order of magnitude as the experiment, indicating the capability of MD simulation in estimating *k*_w_.

The present study conducted by MD simulations clearly shows the proportion of energy consumed by different energy dissipation forms in the nanofriction process to illustrate the wear mechanism. It is believed that these results can promote the application of energy-dissipation analysis in the field of nano-wear and provide guidance for establishing a direct connection between wear and test conditions, material properties, or other factors.

## Figures and Tables

**Table 1:** The wear rate *k*_w_ in the stable sliding stage under different load conditions.

*F*_n_ (nN)	*k*_w_ (10^−4^ mm^3^/Nm)
78	9.75
196	6.13
392	5.58
588	8.47

## Data Availability

The data required to reproduce the results cannot be shared in this stage since it is also a part of another ongoing study.

## Supplementary Materials

The following supporting information can be downloaded at: https://www.mdpi.com/article/10.3390/ma15030893/s1, Figure S1: RDF image of DLC film obtained from OVITO; Figure S2: The evolution of friction force *F*_f_ with *L* under different conditions: (a) *V*_x_ = 1 Å/ps, *T* = 300 K; (b) *F*_n_ = 392 nN, *T* = 300 K; (c) *F*_n_ = 392 nN, *V*_x_ = 1 Å/ps; Figure S3: The sp^2^ ratio of the transfer layer with *L* under different load conditions; Figure S4: The nanoindentation curve of DLC film; Figure S5: The evolution of wear energy *E*_w_ under different conditions: (a) *V*_x_ = 1 Å/ps, *T* = 300 K; (b) *F*_n_ = 392 nN, *T* = 300 K; (c) *F*_n_ = 392 nN, *V*_x_ = 1 Å/ps; Figure S6: The evolution of friction energy *E*_f_ under different conditions: (a) *V*_x_ = 1 Å/ps, *T* = 300 K; (b) *F*_n_ = 392 nN, *T* = 300 K; (c) *F*_n_ = 392 nN, *V*_x_ = 1 Å/ps; Figure S7: The evolution of various energy dissipation forms with sliding distance at *F*_n_ = 78 nN, *V*_x_ = 1 Å/ps, *T* = 300 K; Figure S8: The evolution of various energy dissipation forms with sliding distance at *F*_n_ = 196 nN, *V*_x_ = 1 Å/ps, *T* = 300 K; Figure S9: The evolution of various energy dissipation forms with sliding distance at *F*_n_ = 588 nN, *V*_x_ = 1 Å/ps, *T* = 300 K; Figure S10: Evolution of the proportion of various energy dissipation forms under different *V*_x_ at *F*_n_ = 392 nN, *T* = 300 K: (a) *f*_w_; (b) *f*_p_; (c) *f*_e_; (d) *f*_k_; (e) *f*_t_; Figure S11: Evolution of the proportion of various energy dissipation forms under different *T* at *F*_n_ = 392 nN, *V*_x_ = 1 Å/ps: (a) *f*_w_; (b) *f*_p_; (c) *f*_e_; (d) *f*_k_; (e) *f*_t_; Video S1: 78nN; Video S2: 196nN; Video S3: 392nN; Video S4: 588nN.
