# Supplementary material for: Wear Estimation of DLC Films Based on Energy-Dissipation Analysis: A Molecular Dynamics Study
Source: Materials (Basel). 2022 Jan 25;15(3):893. doi: 10.3390/ma15030893 (PMC8837017; doi:10.3390/ma15030893)
Supplement: Supplementary file 1 [file materials-15-00893-s001.zip › materials-1406611-SM.pdf]

Supplementary

Wear Estimation of DLC Films Based on Energy-Dissipation Analysis: A Molecular Dynamics Study

Zhiyuan Yin [1], Hong Wu [2], Guangan Zhang [3], Chenzhong Mu [4] and Lichun Bai [1,5,*]

[1] Key Laboratory of Traffic Safety on Track, Ministry of Education, School of Traffic & Transportation Engineering, Central South University, Changsha 410075, China; yzy082@csu.edu.cn

[2] State Key Laboratory of Powder Metallurgy, Central South University, Changsha 410083, China; hwucsu@csu.edu.cn

[3] State Key Laboratory of Solid Lubrication, Lanzhou Institute of Chemical Physics, Chinese Academy of Sciences, Lanzhou 730000, China; gazhang@licp.cas.cn

[4] State Key Laboratory of Special Functional Waterproof Materials, Beijing Oriental Yuhong Waterproof Technology Co., Ltd., Beijing 100123, China; mucz@yuhong.com.cn

[5] State Key Laboratory for Strength and Vibration of Mechanical Structures, Xi'an Jiaotong University, Xi'an 710049, China

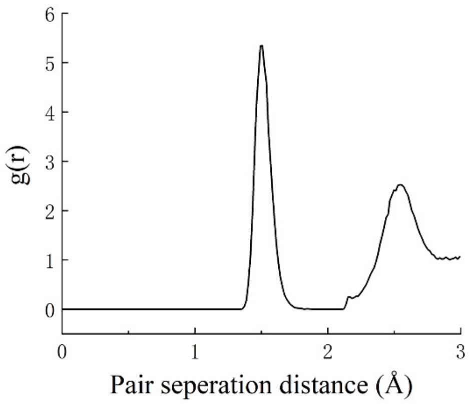

Figure S1. RDF image of DLC film obtained from OVITO.

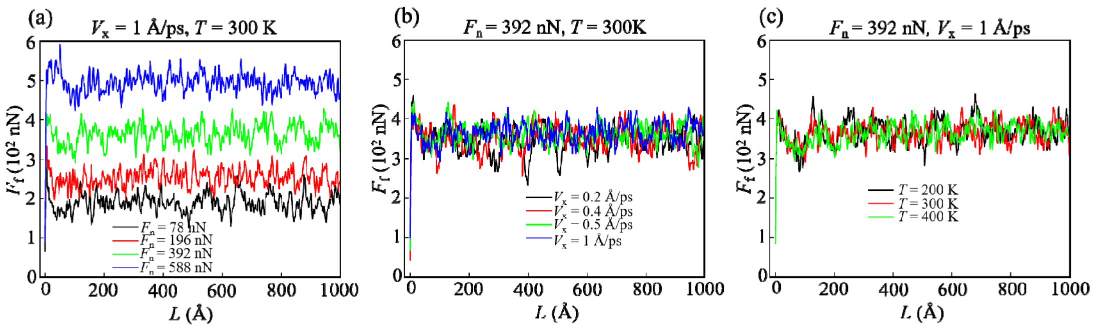

Figure S2. The evolution of friction force F_f with L under different conditions: (a) V_x = 1 Å/ps, T = 300 K; (b) F_n = 392 nN, T = 300 K; (c) F_n = 392 nN, V_x = 1 Å/ps.

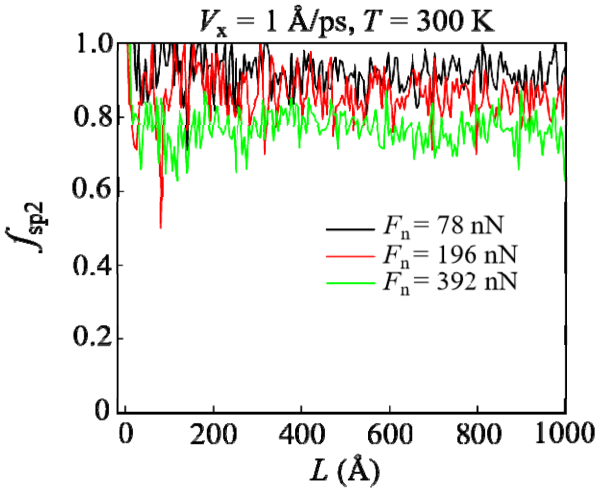

Figure S3. The sp[2] ratio of the transfer layer with L under different load conditions.

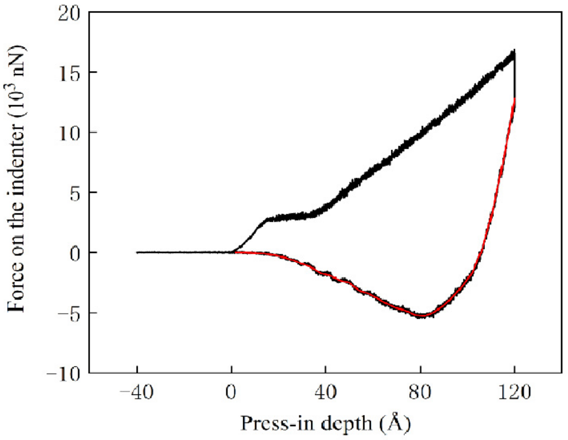

Figure S4. The nanoindentation curve of DLC film.

To ensure the stability of the nanoindentation test, the size of the DLC film is eight times larger than the size in the manuscript. Among them, the test temperature is 300 K, the indentation depth is 120 Å, and the indentation rate is 50 Å/ps. The black curve in the figure is the true curve of the pressing-in-unloading process, and the red curve is the fitting curve of the unloading process.

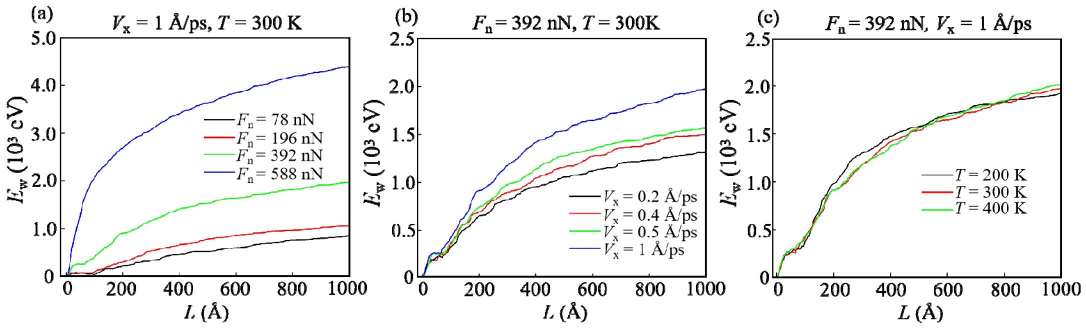

Figure S5. The evolution of wear energy E_w under different conditions: (a) V_x = 1 Å/ps, T = 300 K; (b) F_n = 392 nN, T = 300 K; (c) F_n = 392 nN, V_x = 1 Å/ps.

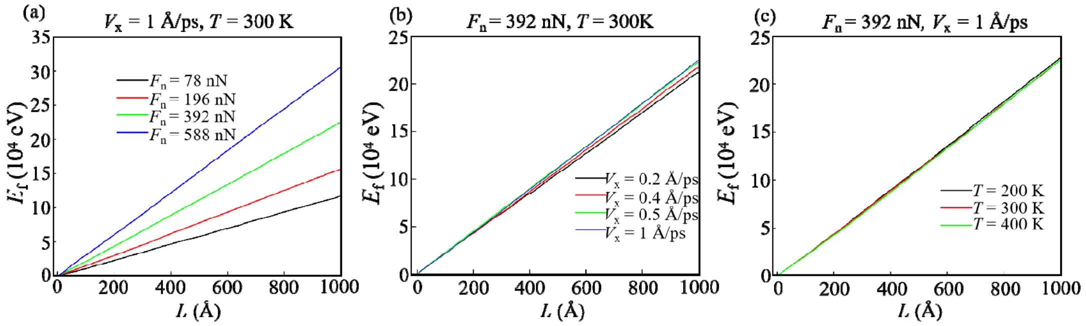

Figure S6. The evolution of friction energy E_f under different conditions: (a) V_x = 1 Å/ps, T = 300 K; (b) F_n = 392 nN, T = 300 K; (c) F_n = 392 nN, V_x = 1 Å/ps.

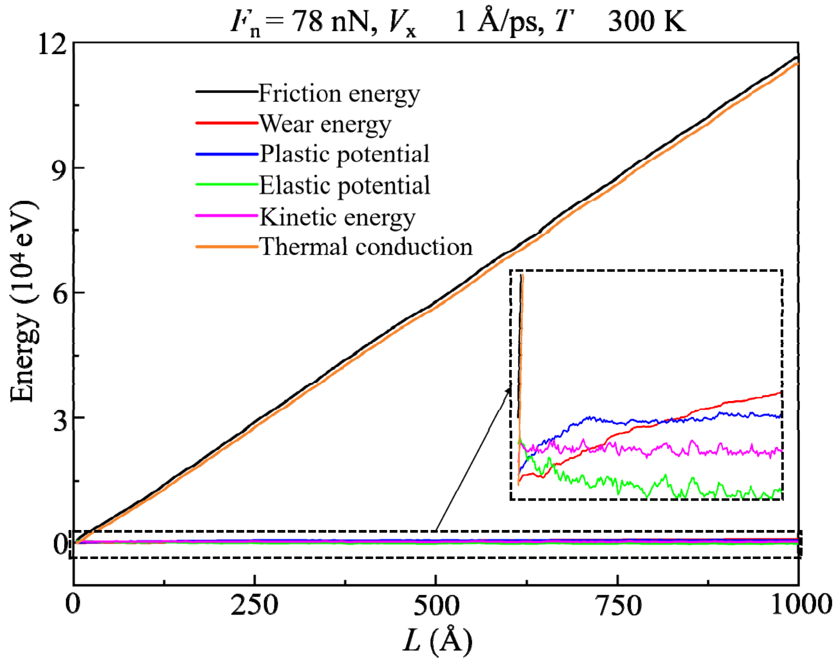

Figure S7. The evolution of various energy dissipation forms with sliding distance at F_n = 78 nN, V_x = 1 Å/ps, T = 300 K.

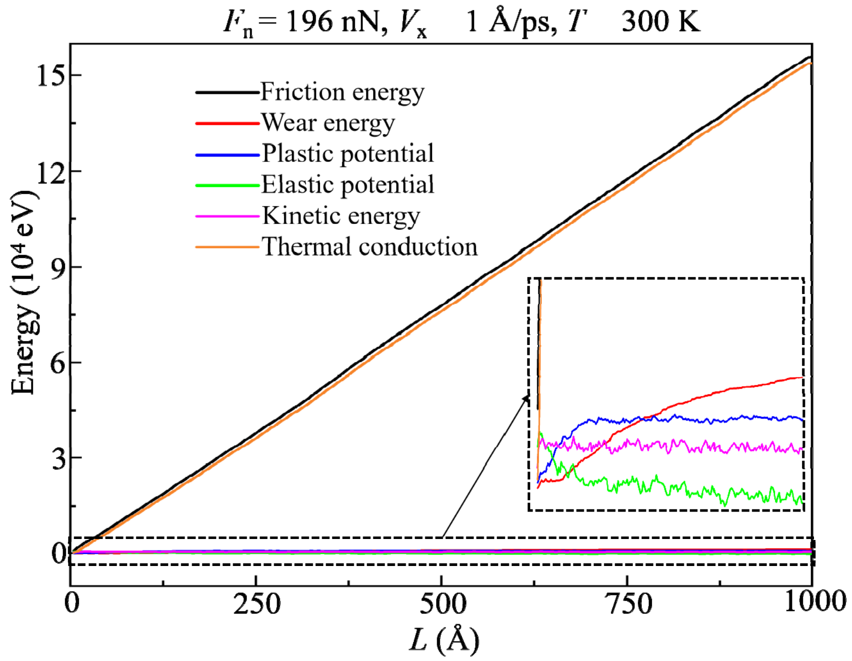

Figure S8. The evolution of various energy dissipation forms with sliding distance at F_n = 196 nN, V_x = 1 Å/ps, T = 300 K.

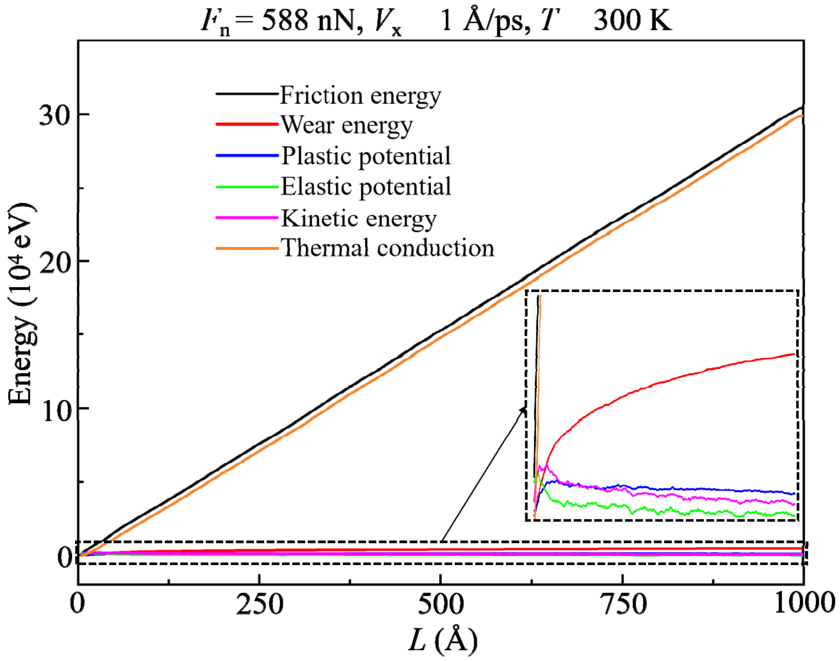

Figure S9. The evolution of various energy dissipation forms with sliding distance at F_n = 588 nN, V_x = 1 Å/ps, T = 300 K.

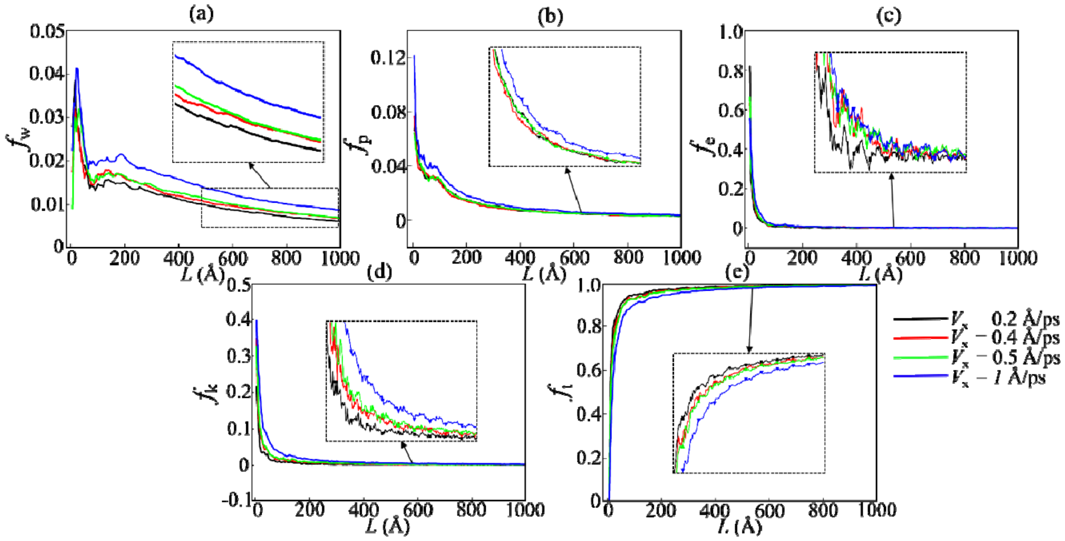

Figure S10. Evolution of the proportion of various energy dissipation forms under different V_x at F_n = 392 nN, T = 300 K: (a) f_w; (b) f_p; (c) f_e; (d) f_k; (e) f_t.

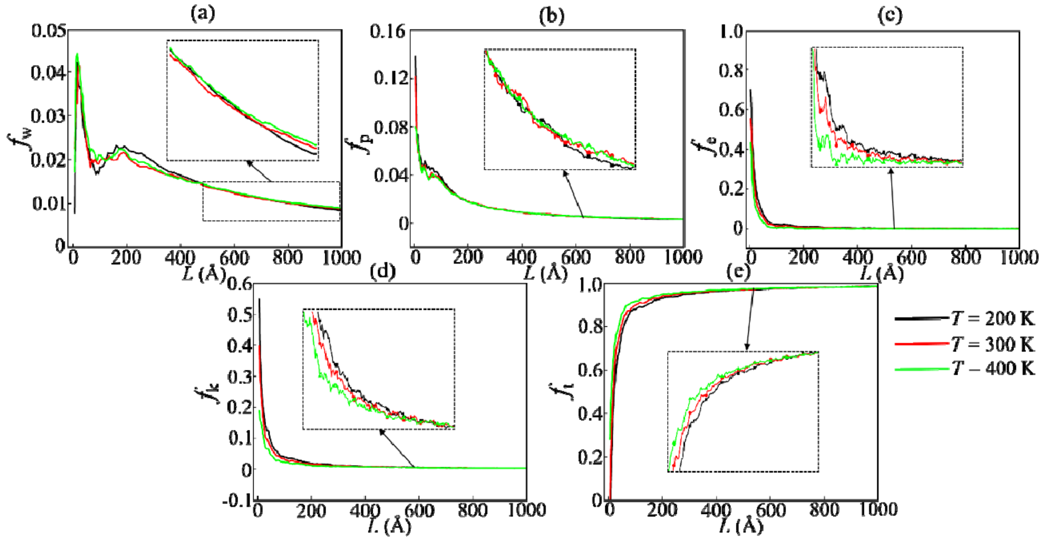

Figure S11. Evolution of the proportion of various energy dissipation forms under different T at F_n = 392 nN, V_x = 1 Å/ps: (a) f_w; (b) f_p; (c) f_e; (d) f_k; (e) f_t. .
